# Editorial: Salutogenesis and public health

**DOI:** 10.3389/fpubh.2026.2006482

**Published:** 2026-10-27

**Authors:** Suvarna Sanjay Sane, Lori Rubenstein Fazzio, Ananda Balayogi Bhavanani, Vijay V. Yeldandi

**Affiliations:** 1 Clinical Trial Unit, ICMR-National Institute of Translational Virology & AIDS Research, Pune, India; 2 Yoga Studies, Bellarmine College of Liberal Arts, Loyola Marymount University, Los Angeles, CA, United States; 3 Institute of Salutogenesis & Complementary Medicine, Sri Balaji Vidyapeeth, Pillayarkuppam, Pondicherry, India; 4 University of Illinois at Chicago, Chicago, IL, United States

**Keywords:** Generalized Resistance Resources (GRRs), health promotion, Indian Knowledge Systems, public health, quality of life, salutogenesis, Sense of Coherence, yoga

This Research Topic, “*Salutogenesis and Public Health*,” brings together nine articles that provide an overview of current salutogenic approaches from individual to societal through their research disseminations. These contributions span clinical, educational, occupational, and community settings.

Together, these articles offer valuable directions for future research in this field of Salutogenesis. Readers who are new to the concept of Salutogenesis are encouraged to begin with the pioneering work of the renowned medical sociologist Aaron Antonovsky. A foundational understanding of Generalized Resistance Resources (GRR) will help readers contextualize his framework. The core concept of Sense of Coherence (SOC) (1), along with the related concept of Generalized Resistance Resources—Resistance Deficits (GRR-RD) (2) provides an important foundation for understanding the articles in this Research Topic.

Public health interventions are typically approached through pathogenic models. The articles in this Research Topic illustrate the important role, a salutogenic approach can offer, in public health (Figure 1). Indian Knowledge Systems revive timeless wisdom, continuously blending ancient insights with modern science to drive future innovations. Oswal et al. have made such an effort by evaluating the effect of ā*nāpāna* meditation on secondary school students. Their findings suggest that this meditation practice helped to increase students' attention span and mental wellbeing as compared to previous year. This intervention has potential of being “implementable classroom GRR.” Another research study in the academic environment by Ke Xu et al. explores the structural relationships between physical activity, self-esteem, grit, and SOC among university students. Their research suggests that physical activity and positive psychological traits strengthen a student's Sense of Coherence and suggests promotion of physical and psychological assets in the universities. They also discuss the limitations of their research and underline the need of probability sampling, repeated measures through longitudinal study design and data collection through wearable devices in future research.

**Figure 1:**
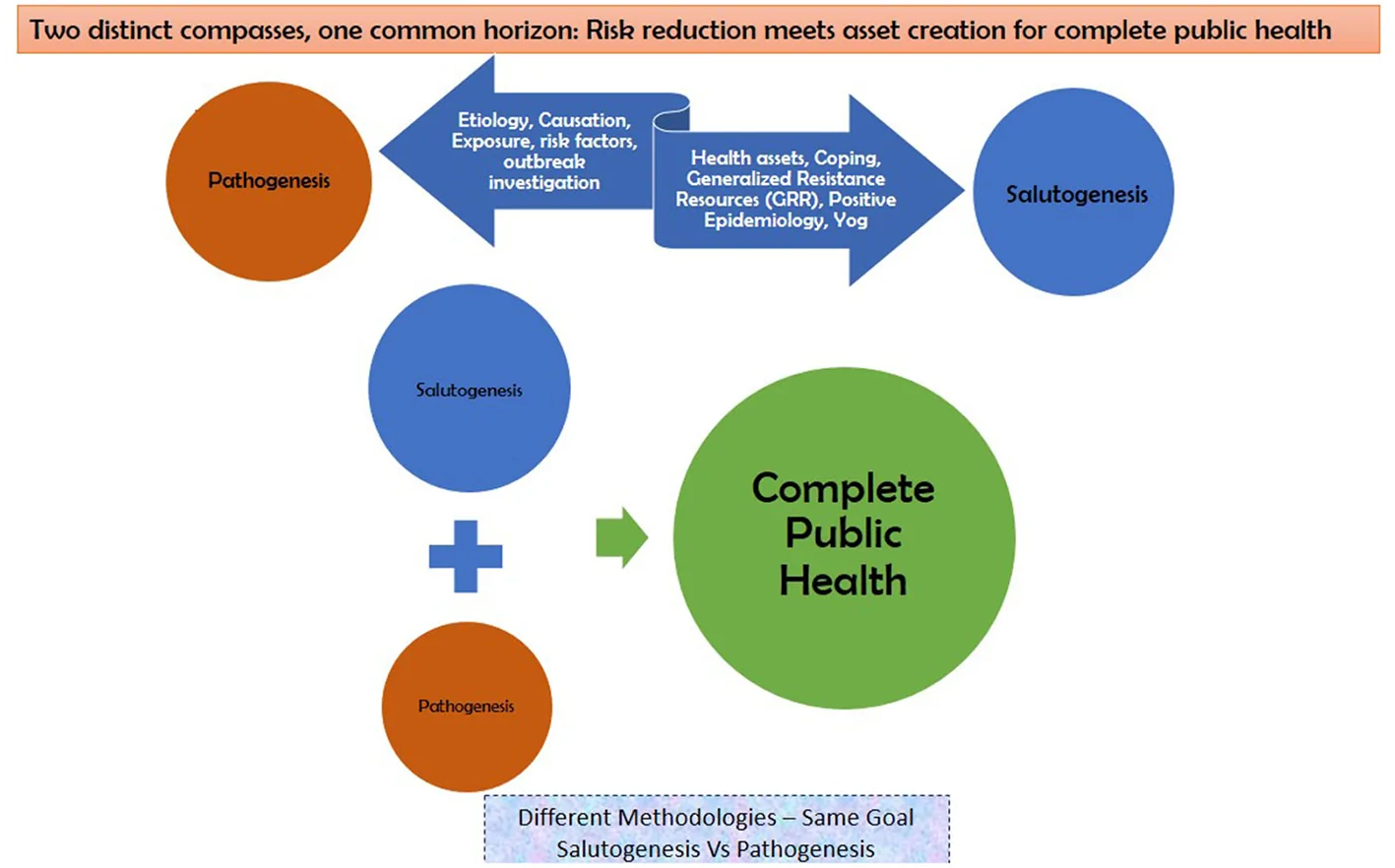
Salutogenesis versus Pathogenesis.

Healthcare access is a universal right, protecting human life from its earliest stages. It is truly the need of the hour to ensure that salutogenic asset-creation is accessible to all. Health care of adolescent girls with intellectual disability (ID) is closely related to comprehensibility and manageability components of the SOC. Anlin Jenisha et al. apply a salutogenic lens to an often-overlooked public health challenge: menstrual and premenstrual problems in adolescent girls with ID. This cross-sectional study calls for inclusive, resource-based public health interventions that empower vulnerable adolescent girls. The authors provide a feasible framework for addressing menstrual health needs within special-school settings. They also recommend to evaluate such integrated strategies for longitudinal effectiveness.

The above-mentioned articles consider the study population in the academic setup. Introducing the concept of health continuum in school-going children especially adolescents, is crucial. Health as continuum in the context of Salutogenesis is discussed in detail along with literature search in a book chapter by Menatti (3).

Although Yoga has been practiced for thousands of years in ancient India, it was reappraised through scientific research for establishment as yogic therapy (4). Zaccari et al. introduce the COMPASS framework and expand on the therapeutic mechanisms of yoga through their hypothesis-and-theory paper. The framework describes how yoga practices can cognitively and physiologically guide individuals from threats to safety and offer a robust salutogenic alternative to pain-management approaches. Tolbaños-Roche et al. evaluate an integrated, comprehensive care model and demonstrate that a yoga-based intervention is strongly associated with enhanced patient wellbeing and high levels of patient satisfaction in intensive psychiatric care. One more original research article in hospital-based setup is done among emergency nurses. Sun et al. investigate the protective effects of perceived social support and moral resilience among emergency department nurses. Their study reveals that these social and moral resources act as crucial buffers, mitigate the impact of moral injury and reduce health-related productivity loss. This highlights the importance of nurturing supportive work environment as organizational GRRs.

This Research Topic encompasses a spectrum of articles, from original research to a systematic review, in various study settings and populations such as schools, hospitals and sports. In their systematic review, Cao et al. in their systematic review of mindfulness-based interventions, indicate that short mindfulness practices can improve sport-related performance and coping mechanisms. The review demonstrates the capacity of interventions to enhance manageability and performance under pressure.

Fazzio et al. examine how *practicing savasana (corpse pose)* can be an accessible practice to invite people to reflect on their mortality, navigate death anxiety, and find meaning in life. This perspective paper suggests that cultivating mortality salience throughout the lifespan may enhance comprehensibility, manageability, and meaning at end of life.

Olson et al. call for “re-storying” traditional health promotion models. They advocate for shifting the locus of control back to communities to foster population sovereignty. This model emphasizes that sustainable health outcomes are achieved when communities are recognized as active, sovereign agents of their own health-generation rather than passive recipients of top-down, pathogenic interventions.

The diverse research compiled in this Research Topic demonstrates that Salutogenesis is a highly versatile and practical framework for modern public health. The evidence is clearly demonstrated through somatic practices in psychiatric wards, mindfulness in sports, supportive social networks in emergency departments, and community-led health promotion models.

Identifying and cultivating health-generating assets builds a stronger, more resilient Sense of Coherence in populations. Public health research worldwide must move beyond conventional disease control and health promotion, embracing salutogenic frameworks, to systematically cultivate factors that create true wellness.
